# Dramatic change in the properties of magnetite-modified MOF particles depending on the synthesis approach

**DOI:** 10.1016/j.heliyon.2024.e27640

**Published:** 2024-03-14

**Authors:** Lyubov Bondarenko, Rose Baimuratova, Marco Reindl, Verena Zach, Artur Dzeranov, Denis Pankratov, Kamila Kydralieva, Gulzhian Dzhardimalieva, Dagmar Kolb, Friedrich E. Wagner, Sebastian P. Schwaminger

**Affiliations:** a Moscow Aviation Institute (National Research University), Moscow, 125993, Russia; b Federal Research Center of Problems of Chemical Physics and Medicinal Chemistry, Russian Academy of Sciences, Chernogolovka, Moscow Region, 119991, Russia; c Division of Medicinal Chemistry, Otto-Loewi Research Center, Medical University of Graz, 8010 Graz, Austria; d Department of Chemistry, Lomonosov Moscow State University, 119991, Moscow, Russia; e Core Facility Ultrastructure Analysis, Center for Medical Research, Gottfried Schatz Research Center, Medical University of Graz, Graz, Austria; f Division of Cell Biology, Histology and Embryology, Gottfried Schatz Research Center, Medical University of Graz, 8010 Graz, Austria; g BioTechMed-Graz, 8010 Graz, Austria; h Department of Physics, Technical University of Munich, James-Franck-Straße 1, 85748 Garching, Germany

## Abstract

Iron-containing metal–organic frameworks are promising Fenton catalysts. However, the absence of additional modifiers has proven difficult due to the low reaction rates and the inability to manipulate the catalysts. We hypothesize that the production of iron oxide NPs in the presence of a metal-organic framework will increase the rate of the Fenton reaction and lead to the production of particles that can be magnetically manipulated without changing the structure of the components. A comprehensive approach lead to a metal organic framework using the example of MIL-88b (Materials of Institute Lavoisier) modified with iron oxides NPs: formulation of iron oxide in the presence of MIL-88b and vice versa. The synthesis of MIL-88b consists of preparing a complexation compound with the respective structure and addition of terephthalic acid. The synthesis of MIL-88b facilitates to control the topology of the resulting material. Both methods for composite formulation lead to the preservation of the structure of iron oxide, however, a more technologically complex approach to obtaining MIL-88b in the presence of Fe_3_O_4_ suddenly turned out to be the more efficient for the release of iron ions.

## Highlights

• Preparation of a metal-organic framework from iron complexes with topology control without toxic organic solvents.
• Formulation of iron oxide with a metal organic framework leads to the destruction of the framework under alkaline conditions.
• Formulation of iron oxide in the presence of a MOF leads to a changed morphology on lamellar structures with large surface.
• The structure of iron oxide is preserved in any approach of tested complex formulation.

## Graphical abstract

**Figure:**
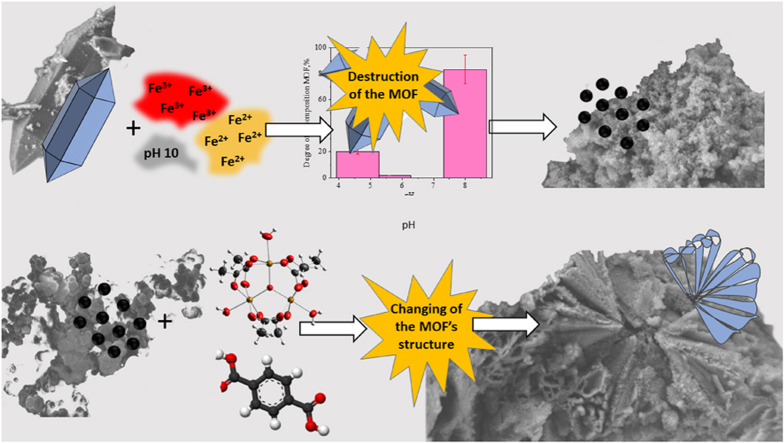

## 1 Introduction

Ferroptosis promises a novel potentially effective treatment strategy for cancer which is based on iron-dependent lipid peroxidation [1],. Many approaches focus on developing effective iron-containing preparations that can controllably induce reactive oxygen species (ROS). These Fenton reactions can cause cell death of cancer cells. The use of metal-organic frameworks (MOF), innovative porous materials consisting of metal ions or their clusters and connected to each other through organic linkers, can be a promising approach to release iron ions [2,3]. Their porosity is expressed by huge surface areas and can reach 7839 m^2^/g [4], and the drug payload can reach up to 25 mass% [[5], [6], [7]]. The undoubted advantage of MOF over other types of porous materials is the possibility of targeted construction of the network topology. This design creates conditions for the specific molecular interaction of a drug and a nanocarrier, and therefore controlling the process of drug delivery [[7], [8], [9]]. However, the use of toxic raw materials in synthesis and the lack of proper purification technology significantly limit the biomedical use of these materials [10].

To obtain MOF, various synthetic approaches have previously been developed based on precipitation reactions at room temperature and under convective heating, including hydrothermal and solvothermal syntheses [11], and alternative routes based on other methods of introducing energy into the system are also used such as electrochemical [12,13], mechanochemical [14,15], microwave [16] and ultrasonic [17] radiation, as well as their combined effects [18]. It is known that when preparing MOFs containing mono- or hetero-metallic polynuclear clusters as an inorganic unit, it is difficult to select the conditions under which the target inorganic unit is formed “in situ” from inorganic salts [11]. For example, the use of ferric chloride and terephthalic acid in traditional solvothermal synthesis to obtain Fe-based MOFs can lead to the production of MIL-101(Fe), MIL-53(Fe), MIL-88B(Fe)), etc. [19]. MOFs with trinuclear inorganic iron units already under visible light undergo direct excitation of Fe–O clusters and induce electron transfer from O^2−^ to Fe^3+^, which subsequently affects the photocatalytic activity [20]. At the same time, the developed porous structure of MOF ensures effective diffusion of reagents to active iron centers. MIL-88B(Fe) is of greatest interest for catalytic transformations, including the Fenton reaction, since it has flexible behavior of the framework in the presence of polar solvents (for example, water), manifested in a change in the volume of its unit cell without loss of topology, which additionally improves the diffusion of reagents to active iron centers [21]. Moreover, electron-rich organic ligands of terephthalic acid typically act as electron donors for the reduction of Fe(III) to Fe(II) [22]. The low toxicity of Fe-based MIL materials was confirmed by both in vitro and in vivo toxicological studies. The safety and structural flexibility have promoted MIL-88B(Fe) to be used for drug delivery [23]. The use of pre-synthesized metal oxo-acetates [M3O(RCOO)6L3] (M = Fe^3+^), including mixed valence, will probably allow us to consider the iron centers of MOFs not only as a structure-forming and transport link, but also as active centers capable of inducing ferroptosis by catalysis Fenton reactions. Coordination vacancy can be created upon the unsaturated Fe ions, which is beneficial for activating H_2_O_2_ [24]. Besides, its zeotype crystal structure endows it with well air, water and solvents resistance. Benefiting from these advantages, various MIL(Fe)-MOFs including MIL-88B [25], NH_2_-MIL-101Fe [26], and NH_2_-MIL-88B [27] have seen reports in heterogenous Fenton-like processes. But the results are inconsistent. For instance, for NH_2_-MIL-101Fe, HO_2_· and·O^2−^ rather than ·OH were reported to be the main ROS to degrade pollutants [28]. Dissolved iron ions in the solution could also react with H_2_O_2_ and generate ·OH but with slower rate. For NH_2_-MIL-88B, it was reported that ·OH and ·O^2−^ are the dominant radicals in degradation [29]. Non-targeted ferroptosis induction can interfere with iron homeostasis and cause excessive ROS production, which can affect the immune system and cause neurodegenerative conditions such as Huntington's and Parkinson's disease, heart failure, and leukemia [[30], [31], [32], [33], [34]]. Therefore, it would be promising to create materials that would have synergistic properties: magnetic control and high catalytic activity of the Fenton reaction. There are different strategies to improve MIL-88b properties. Ye et al. [35] formulated MIL-88B(Fe)/Fe_3_S_4_ hybrids with numerous and durable unsaturated iron sites, and S^2−^ ions acting as electron donors that enhanced the Fe(II) recycling in electro-Fenton (HEF) treatment of organic micropollutants at pH 7.0. Despite the quickest complete removal of trimethoprim at mild pH, only requiring 45 min, this particles cannot be controlled by magnetic field that which complicates their separation. Usman Akbar et al. [36] suggested to use multifunctional bimetallic (FeCo) bi-MIL-88B-FC MOFs modified with folic acid-conjugated chitosan as drug delivery systems (DDS) for targeted delivery of 5-Fluorouracil, but finally obtaining a bimetallic iron-cobalt alloy is time-consuming and cost process.

In our study we have selected Fe_3_O_4_ nanoparticles (NPs) as a magnetic material attached to the surface of the MOF or embedded inside the MOF. After introduction of the Fe_3_O_4_ NPs into MIL-88B(Fe), the magnetic MOFs should have superparamagnetic properties, so that the particles can be easily separated from the reaction mixture using an external magnetic field and also redispersed in the liquid phase after removal of the external magnetic field [37].

The aim of this work is to formulate iron-containing mesoporous materials that have the ability to induce ROS in high concentrations under model conditions. To obtain MIL-88b, a rational design for the preparation of MOF was used, which consisted of first preparing a complex compound that contains the required lattice unit (in this case, it is pre-synthesized trinuclear iron acetate), then the acetic acid residues were exchanged for the corresponding linkers that will connect these units in the lattice [38,39]. The advantage of this approach is the ability to control the topology of the resulting metal-organic framework, and, in general, the phase purity of the reaction product [40]. In search of the most gentle approaches to the synthesis of magnetite-modified MOF, we propose two production routes: formulation of iron oxide in the environment of pre-synthesized MOF (MOF-Fe_3_O_4_) and formulation of MOF in the presence of pre-synthesized magnetite (Fe_3_O_4_-MOF). Thus, we have studied the microstructure, physicochemical characteristics, and the release of iron ions from MOF, Fe_3_O_4_-MOF and MOF-Fe_3_O_4_ samples as an important Fenton-parameter for ferroptos initiation.

## 2 Materials and methods

### 2.1 Chemicals and reagents

Ethanol and N, N-dimethylformamide (DMF) were purchased from RUSKHIM Chemicals Company (Moscow, Russia) and purified by distillation. Iron(III) chloride (FeCl_3_·6H_2_O), sodium acetate (CH_3_COONa·3H_2_O), Iron(II) chloride (FeCl_2_·4H_2_O) and terephthalic acid (C_6_H_4_(CO_2_H)_2_) was classified of at least as reagent grade and purchased from Sigma Aldrich. The water used in all experiments was deionized.

### 2.2 Preparation of MOF

**Fig. 1:**
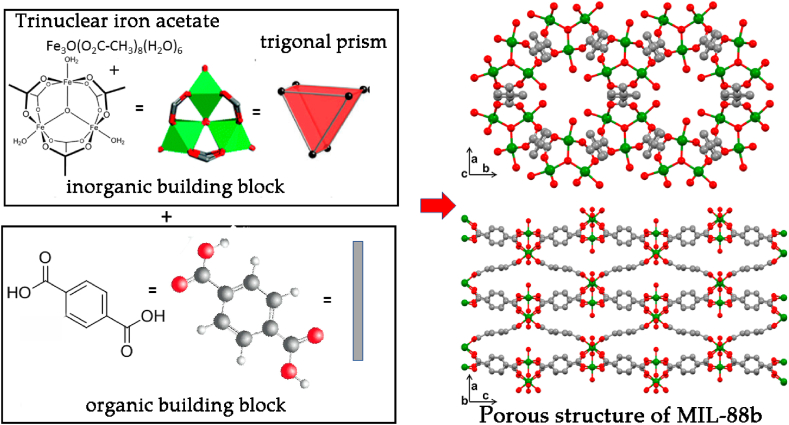
Scheme of a metal-organic framework MIL-88b. On the left is secondary building units (SBUs) of MIL-88b. The ditopic terephthalate linker had abstracted as a rod, the Fe_3_O(-CO_2_)_6_ SBU as trigonal prism, and show their assembly into asc net (six-connected topology in which the node is a trigonal prism [42]). Hydrogen atoms have been left out for clarity. Fe:green, O: red, C:grey. (For interpretation of the references to colour in this figure legend, the reader is referred to the Web version of this article.)

The preparation of metal-organic framework (MOF) was carried out in two stages (Fig. 1). At the first stage complex compound which contains the necessary lattice node was synthesized. There is a trinuclear acetate block with the empirical formula [Fe_3_O(CH_3_COO)_6_(H_2_O)_3_]Cl·6H_2_O (Fe_3_OAcetate), then ligands of this node have been replaced by a terephthalic acid linker, which connected these nodes in the lattice.

Trinuclear acetate block synthesized according to the procedure described in the literature [41]. For synthesis FeCl_3_·6H_2_O (24.33 g, 0.09 mol) was dissolved in 30 ml of hot water (50 °C). Sodium acetate (24.48 g, 0.18 mol) was dissolved separately in 30 mL of hot water (50 °C). The resulting solutions were mixed together and continued stirring at 50 °C for 25 min. The solution was left to cool in the air overnight for crystallization. After a few days, an orange-red powder was isolated on a Buechner funnel and washed with a small amount of cold ethanol. Then the precipitate was air dried to a constant mass for several days. Yield: 16.94 g (77%). Found (%): C, 19.5; H, 5.1; Fe, 23.1. Calculated for [Fe_3_O(CH_3_COO)_6_(H_2_O)_3_]Cl·6H_2_O (wt%): C, 19.60; H, 4.93; Fe, 22.78; Cl,4.82; IR (KBr -tablet), *ν*/cm^−1^: 3523 *ν* (O–H); 2929 *ν*(CH); 1601, 1520 and 1444 νCOO; 1028; 664,599 *ν* (Fe–O).

For MOF synthesis terephthalic acid (5 g, 0.03 mol) and the Fe_3_OAcetate (7.35 g, 0.01 mol) were dissolved in 100 ml of DMF under stirring on a magnetic stirrer. The resulting mixture was boiled for 6–8 h under reflux at 153 °C until a dark red precipitate formed. The precipitate was isolated in a Buechner funnel, washed several times with fresh DMF, absolute ethanol and dried in vacuo (10^−3^ Torr, 80 °C, 10 h). Yield: 6.82 g (89%).%). Found (%): C, 36.72; H, 2.64; Fe, 21.6. Calculated for [Fe_3_O(C8H4O4)3(H_2_O)_3_]Cl (wt%): C, 37.77; H, 2.37; Fe, 21.89; Cl,4.63; IR (KBr -tablet), *ν*/cm^−1^: 3420 *ν* (O–H); 2925 *ν*(CH); 1659,1578,1504 and 1391 νCOO; 748; 548 *ν* (Fe–O).

### 2.3 Preparation of the MOF-Fe_3_O_4_

In the first approach, magnetite NPs were synthesized in the presence of MOF by the Elmore reaction (eq. (4)) [43]:

(4) $$2\;\text{Fe}Сl_{3}+{\text{FeCl}}_{2}+8\;\text{KOH}\;\to \;{\text{Fe}}_{3}O_{4}↓+8\;\text{KCl}+4\;H_{2}O\text{,}$$

For this purpose, 10.08 g (0.0373 mol) of FeCl_3_·6H_2_O crystalline hydrate and 3.706 g (0.01864 mol) of FeCl_2_·4H_2_O crystalline hydrate were dissolved in 200 mL of distilled H_2_O in an argon atmosphere in the presence of 8 g of MOF. The solution was heated to 60 °C. With vigorous stirring, a 10% KOH solution was added to raise the pH to 10. The resulting MOF-magnetite precipitate was separated from the solution on a Büchner funnel by washing with pre-boiled hot (60–80 °C) distilled water, the sample was dried in vacuum (50°С, 12 h). Yield: 4.68 g (39%). Found (%): C, 0.89; H, 0.96; Fe, 64.5. IR (KBr-tablet), *ν*/cm^−1^: 3400 *ν* (O–H); 1619 and 1384 *ν* (COO); 1056; 548 *ν* (Fe–O).

### 2.4 Preparation of the Fe_3_O_4_-MOF

In the second approach, MOF was synthesized in the presence of pre-synthesized Fe_3_O_4_ NPs. For this, on the first step magnetite was obtained by the Elmore reaction according to the 2.3 part in the absence of MOF. Further to 4 g of magnetite a solid terephthalic acid (5.90 g, 0.035 mol) and the Fe_3_OAcetate (8.70 g, 0.0118 mol) were added. The pH value was adjusted to 9 using a 10 M ammonia solution. The mixture was kept stirring on a mechanical stirrer for 30 min. The precipitate was purified in the same way as described in the 2.2. Yield: 9.69 g (80 %). Found (%): C, 11.74; H, 2.72; Fe, 42.9. IR (KBr -tablet), *ν*/cm^−1^: 3414 *ν* (O–H); 2930; 1632,1558, and 1385 *ν* (COO); 1017; 583 *ν* (Fe–O).

### 2.5 Characterization of samples

X-ray diffraction (XRD) was performed in transmission geometry with a Stadi-P from STOE & Cie GmbH, Germany equipped with a MoKα source (λ = 0.7093 Å). The full width at half maximum (FWHM) of the all reflections was used for particle size determination with the Scherrer equation (Suppl, eq. S1). In order to quantify oxidation progress the (440) reflection was fitted with Voigt functions in Origin 2019 Pro (Suppl., eq S2).

The 440 reflection was fitted with a Voigt function with the software Origin in order to analyze the content of magnetite and maghemite based on the areas (A) of the respective reflections considering the following Equation (5):

(5) $${\text{Fe}}_{3}O_{4}\;\left(\%\right)=100\frac{A\;\left({Fe}_{3}O_{4}440\right)}{A\left({Fe}_{3}O_{4}440\right)+A\left(\gamma {Fe}_{2}O_{3}440\right)}$$

Mössbauer spectra were measured in transmission geometry using an electromechanical velocity drive with a sinusoidal velocity waveform (Halder Elektronik, Germany) and a 25 mCi source of ^57^Co in a rhodium matrix. The spectrometer was calibrated against α-Fe at ambient temperature. The 14.4 keV γ-rays were detected with a Kr proportional counter with single-channel analyzer windows set to the 14.4 keV photo-peak and the escape peak. For measurements at 4.2 K, the ^57^Co-source and the absorber were cooled in a liquid helium bath cryostat. The Mössbauer spectra were approximated using the SpectRelax 2.8 software (Lomonosov Moscow State University, Russia). Isomeric shifts were converted relative to α-Fe at room temperature.

Raman spectroscopy was performed with a Raman Senterra spectrometer from Bruker Optics, Germany. Suspension drops had been deposited on microscope slides and dried under nitrogen atmosphere before spectra were recorded. A 488 nm laser was used and the laser power was reduced by optical filters to 0.1 mW for each measurement. In order to quantify the progress of oxidation, the A_1g_ band between 600 and 750 cm^−1^ was fitted with Voigt functions in Origin 2015 Pro.

The bands at 660 cm^−1^ and 710 cm^−1^ of MOF modified NPs (Fig. 6) were fitted with Voigt functions in Origin and used for magnetite content analysis (OriginLab Corporation, Wellesley, Massachusetts, US) (Fig. 6 c, d). The deconvolution was processed with Equation (6) in order to obtain the magnetite share [44].

(6) $${\text{Fe}}_{3}O_{4}\;\left(\%\right)=100\left(1-\frac{A\left(710\right)}{A\left(660\right)}\right)$$

The identification of functional groups and additional information on the type of coordination was carried out using FTIR spectroscopy (Spectrum 2, PerkinElmer, USA) with a spectral resolution of 2 cm^−1^ for the wavenumber range of 4000–350 cm^−1^ using a platinum attenuated total reflection device.

Scanning electron microscopy (SEM). Double sticky tape was placed on SEM stubs, and the particles were placed onto the stubs. Next, the samples were sputtered with a gold–palladium mixture at 10 nm thickness using a Bal-Tec SCD500 sputter coater. The particles were visualized using a Sigma 500 VP FE-SEM operated at 3 kV with a secondary electron detector.

Transmission electron microscopy (TEM). The morphology of the particles was evaluated using a Tecnai G2 20 transmission electron microscope (FEI Company, USA) operated at an accelerating voltage of 120 kV. Therefore, all samples were dissolved in distilled water using an ultrasonic processor (Fisherbrand, USA) at 75 % amplitude until a turbidity was visible by naked eye. 10 μL of each suspension was applied to carbon-coated copper grids (size 200 mesh) which had been pre-treated in a glow discharge cleaning system (PELCO Inc., USA). After an incubation for 1 min at room temperature, excessive liquid was gently removed by means of a filter paper. The TEM specimens were mounted in a TEM holder and analyzed subsequently. Images were acquired using a BM-Ultrascan 1000P CCD camera.

The magnetic properties of MNPs dry powders were characterized with a Vibrating Sample Magnetometer Lake Shore (Lake Shore Cryotronics, Westerville, OH, USA) at 300 K.

Textural characteristics of MNPs were measured using nitrogen adsorption/desorption isotherms at 77 K (liquid N_2_) using a Quadrasorb SI setup (Quantachrome, USA) by the static volumetric method; samples were degassed by heating at 150 °C for 4 h under vacuum prior to analysis.

## 3 Results

### 3.1 Characterization

**Fig. 2:**
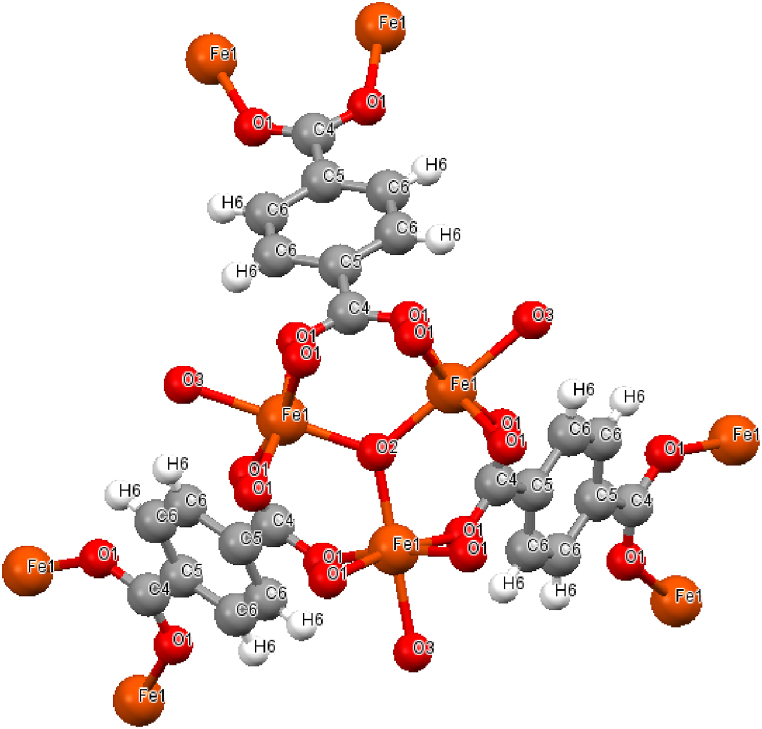
Structure of the elementary unit of the coordination polymer Fe_3_O(C_8_H_4_O_4_)_3_(H_2_O)_3_]Cl according to CCDC 2088535.

**Fig. 3:**
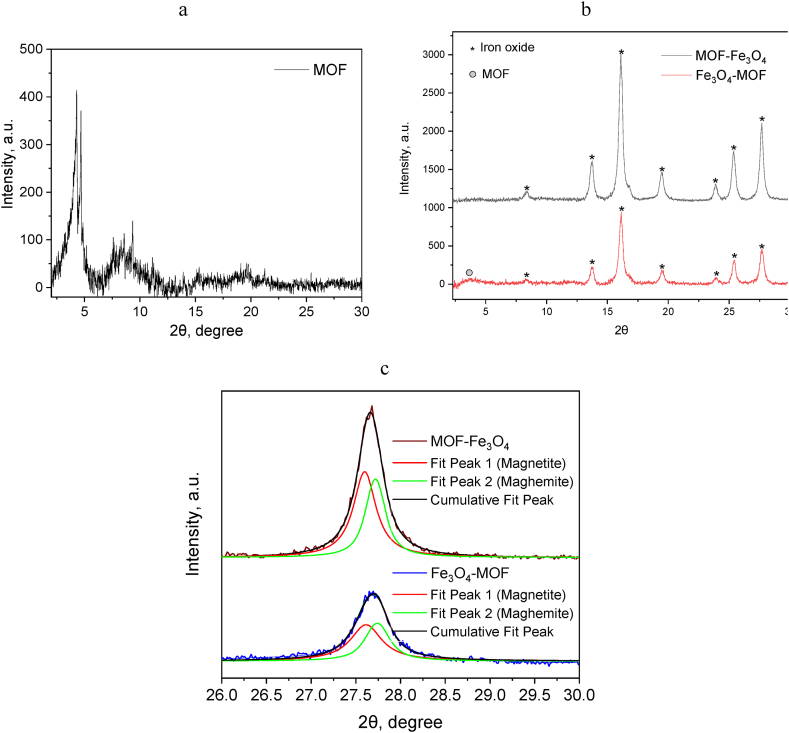
XRD for MOF (a), MOF modified with Fe_3_O_4_ (b) and deconvolution of (440) peak of modified MOF (c) (R^2^ 0.99 and 0.98 for MOF-Fe_3_O_4_ and Fe_3_O_4_-MOF, respectively) (MoKα source (λ = 0.7093 Å).

The purity of the resulting coordination polymer was confirmed by XRD (Fig. 3) and elemental analysis. To confirm the compliance of the obtained sample with the proposed structure, a diffraction pattern is presented in comparison with the calculated CCDC 2088535 (Suppl., Fig. S1) [45]. The coordination polymer is a MIL-88b-type framework having a hexagonal topology, in which the three-core iron cluster acts as a six-connected node with trigonal prism geometries. The elementary unit is a cluster compound, the metal centers of which are equivalent and each of them is octahedral bonded to six oxygen residues of terephthalic acid (Fig. 2).

It should be noted that the XRD profile of MOF after purification of dimethylformamide molecules with ethyl alcohol changes (Fig. 3a). This phenomenon is sometimes observed for flexible MOFs and called as « guest-responsive structural behavior » [46]. In our system, it was shown that the coordination polymer exhibits a change in the orientation of the metal cluster and phenyl rings due to their rotation around the O–O axis of the carboxylate groups (knee-like axis of rotation) [47].

The crystalline structures of the MOF modified with Fe_3_O_4_ were identified with Rietveld refinement using Profex software (Fig. 3b, Suppl. Fig. 2S). The value goodness of fit for both samples was ∼1.2. The lattice constants calculated from XRD are 8.378 Å and 8.373 Å for MOF-Fe_3_O_4_ and Fe_3_O_4_-MOF, respectively. According to ICDD-PDF 19–629, the characteristic peaks at 8.3, 13.7, 16.1, 19.5, 23.9, 25.9 and 27.7° corresponds to Fe_3_O_4_ phase in both samples. The broad peak at 4° in the sample can be attributed to MOF as the presence of carbon structure is further confirmed using FTIR spectroscopy and elemental analysis. The intensity of the peaks in the diffraction pattern indicates a higher content of the iron oxide phase in the MOF-Fe_3_O_4_ sample compared with the Fe_3_O_4_-MOF, where the MOF was obtained in the presence of magnetite. The results are consistent with the elemental analysis indicated 64.5 % and 42.9 % of Fe for MOF-Fe_3_O_4_ and Fe_3_O_4_-MOF respectively.

The crystallite size was estimated from XRD data with the Scherrer equation (Suppl., eq. S1). We used a spherical shape factor (0.89) and obtained smaller particle diameters than those measured manually on TEM images which is consistent with literature [80]. The diameter of NPs calculated by the Scherrer equation is 13.2 ± 3.8 nm for MOF-Fe_3_O_4_ and 12.6 ± 5.4 nm for Fe_3_O_4_-MOF, respectively.

Using XRD data-based approach from Kim et al. [48], which tried to differentiate between the distorted spinel of maghemite and the inverse spinel of magnetite in a powder XRD, we have deconvoluted the (440) reflection (Fig. 3c), which is a superposition of peaks at 27.59 ± 0.03° and 27.71 ± 0.03° for MOF-Fe_3_O_4_ and 27.61 ± 0.20° and 27.74 ± 0.11° for Fe_3_O_4_-MOF, respectively.

If we assume that the reflection angle corresponds to magnetite, and the peak larger reflection angle corresponds to maghemite, then the MOF-Fe_3_O_4_ contains 57.6% Fe_3_O_4_ and Fe_3_O_4_-MOF – 56.9% of Fe_3_O_4_, which indicates partial oxidation of magnetite during the synthesis of MOF.

**Fig. 4:**
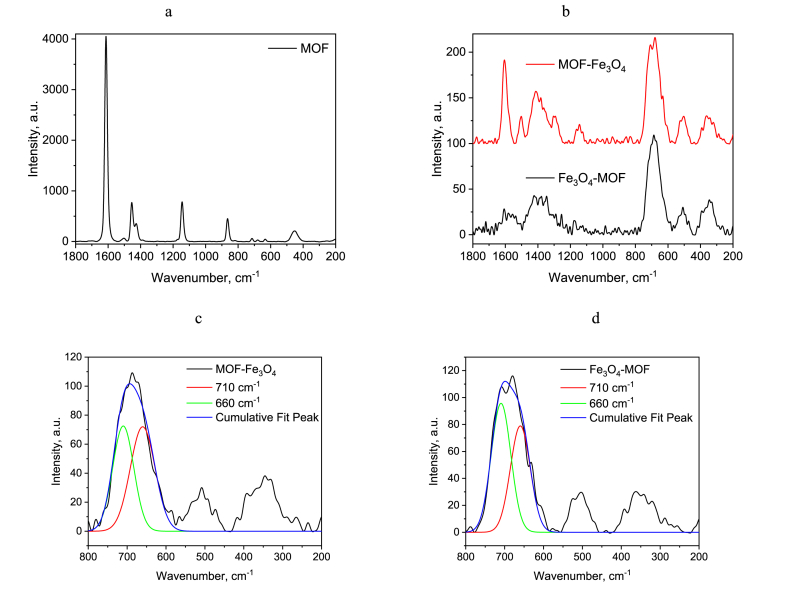
Raman spectra of samples MOF (a) and MOF modified with Fe_3_O_4_ (b), which illustrate the deconvolution fitting of MOF-Fe_3_O_4_ (c) and Fe_3_O_4_-MOF (d).

Raman spectroscopy data allowing to recognize both MOF structural groups and iron oxide phase change are presented on Fig. 4. As showed on Fig. 4a, the bands of Raman spectra of MOF at 1612 cm^−1^ were assigned to the COO^−^ asymmetric stretching vibration [49,50]. The most prominent band (1612 cm^−1^) is retained for samples modified with iron oxide, and a significant decrease in its intensity is associated with a decrease in the concentration of carbon and MOF in the end according to elemental analysis. The band at 634 cm^−1^ is assigned to the in-plane bending of the carboxylate group OCO [51]. The medium band at 450 cm^−1^ is assigned to the metal–oxygen bond [52]. Bands at 1427 cm^−1^ and 1454 cm^−1^ can be attributed to symmetric COO^−^ stretching vibrations in DMF and 1144 cm^−1^ – to vibration of CH-group [50,53]. Previously, Schwaminger et al. [44,54] showed the interpretation of Raman spectra for iron oxide phase and great advantage of the analysis of Raman spectra for non-crystalline and extremely small particles. The oxidation of the divalent iron ions can be verified by the Raman mode (A_1g_) near 660 cm^−1^. For maghemite this mode is split into two equal components around 660 and 710 cm^−1^ [55,56]. According to Ref. [57], the second mode refers to changes in the distance between Fe ions and O ions.

**Fig. 5:**
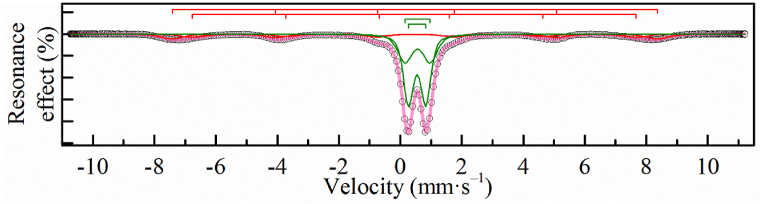
Mössbauer spectrum at 4.2 K of a MOF sample.

**Fig. 6:**
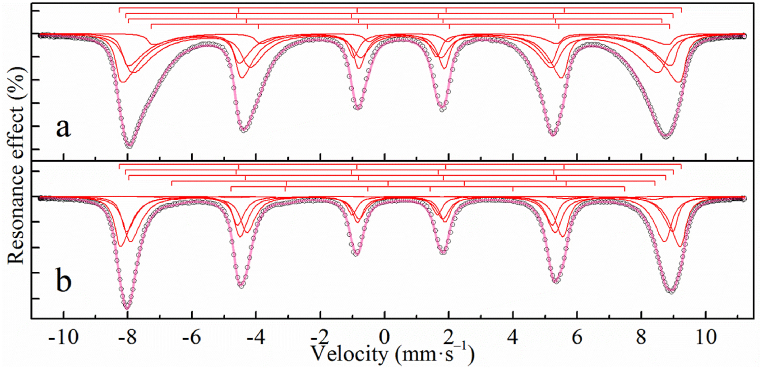
Mössbauer spectra at 4.2 K of Fe_3_O_4_-MOF (a) and MOF-Fe_3_O_4_ (b) samples.

According to the calculations, the magnetite content in the magnetite-maghemite mixture is 0% for MOF-Fe_3_O_4_ and 14.4% for Fe_3_O_4_-MOF, which a bit differs from XRD data, but is in good agreement with Mössbauer data (Table 1, Fig. 5, Fig. 6).

**Table 1:** ^57^Fe Mössbauer parameters for the samples at 4.2 K.

Sample	№	δ^a^, mm/s	Δ, mm/s	Γ_exp_, mm/s	H_eff_, kOe	α = KV/k_B_T	S, %
MOF	1	0.50	−0.06	0.87	489.3		19.2
	2	0.46	**−**0.02	1.01	448		15.1
	3	0.54	0.58	0.38			35
	4	0.57	0.77	0.71			30.9
Fe_3_O_4_-MOF	1	0.52	−0.03	0.34	543.0	8.20	36
	2	0.40	0.14	0.32	528.9		22
	3	0.42	−0.17	0.54	515.5		35
	4	0.78	0.06	0.31	501.1		7.3
MOF-Fe_3_O_4_	1	0.51	−0.03	0.39	542.7	20.7	36.3
	2	0.40	0.14	0.34	529.4		23.1
	3	0.46	−0.11	0.46	518.9		37
	4	1.10	−0.41	0.60	467.0		2.6
	5	0.90	0.90	0.60	380.0		1.3

a δ — the isomeric shift, Δ — the quadrupole splitting, Γ_exp_ — the linewidth, H_eff_ — the hyperfine magnetic field, S — the relative area of the subspectrum, α – the quotient of particle anisotropy energy to thermal energy as (7).

The Mössbauer spectrum at 4.2 K for the MOF sample is an intense symmetrical doublet against the background of a low-intensity sextet, somewhat broadened towards the inner part of the spectrum (Fig. 5). The experimental spectrum can be satisfactorily described by a model containing a superposition of two nested doublets with similar isomeric shifts, but differing in quadrupole splitting, and two nested sextets, which differ mainly in the values of the hyperfine magnetic field (Table 1). The hyperfine parameters of the doublets correspond to Fe^3+^ atoms in the high-spin state and octahedral environment [[61], [62], [63]]. The presence of sextets in the spectra may be due to the presence of impurities in the form of MOF hydrolysis products [64,65].

The Mössbauer spectra of the Fe_3_O_4_-MOF and MOF-Fe_3_O_4_ samples at 4.2 K are asymmetric sextets with distorted resonance line profiles due to a noticeable broadening towards the inner region of the spectrum (Fig. 6). In this case, the width of the resonance lines and their distortions are noticeably higher in the case of the spectrum for the Fe_3_O_4_-MOF sample compared to MOF-Fe_3_O_4_. This indicates the manifestation by the particles of the phenomenon of superparamagnetism, which is characteristic of nanosized particles, and the particle sizes of the Fe_3_O_4_-MOF sample are smaller than the particle sizes of the MOF-Fe_3_O_4_ sample [66,67]. The features of the Mössbauer spectrum of the MOF-Fe_3_O_4_ sample include the presence of weak but clearly expressed resonant absorptions in the regions of −3 and 4 mm/s (Fig. 6) characteristic of magnetite, which can be caused by sextets of Fe^2+^ atoms in octahedral positions [68]. For the spectrum of the Fe_3_O_4_-MOF sample, such features cannot be detected, possibly due to the high width of the resonance lines. Both spectra can be satisfactorily described by a set of 4 or 5 sextets (Fe_3_O_4_-MOF and MOF-Fe_3_O_4_, respectively) whose profile is set within the many-state superparamagnetic relaxation model [69,70] (Table 1). In this case, within the framework of each sample, the relaxation spectra were related by a single relaxation time and the quotient of the magnetic anisotropy energy of the particles to the thermal energy:

(7) $$\alpha =K\;V\;/\;k_{B}\;T\text{,}$$

where K is the magnetic anisotropy constant, V is the volume of the magnetic domain, k_B_ is the Boltzmann constant, T is the temperature [70]. On the whole, the obtained parameters (Table 1) agree with similar hyperfine parameters for low-temperature spectra of magnetite [68,71]. Analyzing the ratio α(Fe_3_O_4_-MOF)/α(MOF-Fe_3_O_4_) and assuming that the magnetic anisotropy constants for the samples are equal, one can easily show that the ratio of the linear dimensions of the magnetic domains is: d(Fe_3_O_4_-MOF)/d(MOF-Fe_3_O_4_) = 0.73. Both spectra indicate no or just a very small amount of divalent iron ions. Assuming similarly [68] that subspectra 4 and 5 refer to Fe^2+^ atoms in the octahedral positions of spinel, and assuming that the areas of the subspectra are proportional to the content of iron atoms in the corresponding states, the ratio of iron ions can be calculated as [72]:

(8) $$X_{ms}=\frac{{\text{Fe}}^{2+}}{{\text{Fe}}^{3+}}=\frac{S4+S5}{S1+S2+S3}\text{,}$$

where S# − area of subspectrum #. From the resulting relationship it is easy to estimate the nonstoichiometry parameter of magnetite - δ [73]:

(9) $$\delta =\frac{1-2X_{ms}}{3+2X_{ms}}\;\text{.}$$

Thus, from the experimental data of Mössbauer spectroscopy, the nonstoichiometry parameter δ for Fe_3-δ_O_4_-MOF and MOF-Fe_3-δ_O_4_ can be estimated as 0.2668 ± 0.0024 and 0.2982 ± 0.0009, respectively. This indication is in good agreement with the Raman spectra which also indicate an oxidation of the iron oxide particles.

**Fig. 7:**
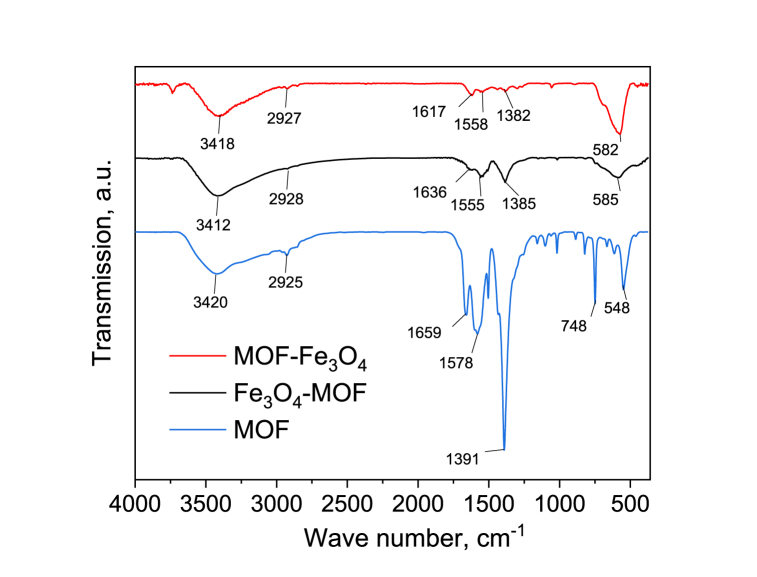
FTIR spectra for MOF, MOF-Fe_3_O_4_ and Fe_3_O_4_-MOF.

Fourier transform infrared spectrophotometry (FTIR) was used also for quantification of magnetite and maghemite phase. In accordance with the IR spectroscopy data presented in Fig. 7, MOF demonstrates broad absorption bands in the range of 2800–3600 cm^−1^, which correspond to the stretching vibrations of OH-groups of both physically adsorbed and crystallized water molecules.

Stretching vibrations of the = C–H group in terephthalic acid molecules appear in the spectrum at 2950-2640 cm^−1^ with a maximum intensity at 2925 and 2855 cm^−1^, which is also absent on the surface. Asymmetric and symmetric stretching vibrations of the COO^−^ carboxyl group are observed at 1580-1565 and 1444-1378 cm^−1^. Since the nature of carboxylate coordination can be judged from the difference between ν_as_(COO^−^) and ν_s_(COO^−^), it is assumed that the carboxylate ion is coordinated with the metal cation in the bidentate coordination mode (Δν ≤ 187 cm^−1^) according to the literature data [52,58]. The metal-oxygen (Fe–O) vibration band is present in the region of 548 cm^−1^ and, according to compassion of bond's squares, 47.6% of Fe–O are located on the surface.

The functionalization of MOF leads to a decrease in the intensity of the bands related to the presence of water in the samples (region ∼3400 cm^−1^) by 20% sequentially when obtaining MOF in the presence of magnetite and by 50% when obtaining magnetite in the presence of MOF (Fig. 7). In addition, both approaches to the preparation of the nanocomposite led to a decrease in the intensity of the C=O bands by 85% and 92% for Fe_3_O_4_-MOF and MOF-Fe_3_O_4_, respectively, in comparison to MOF, which is confirmed by elemental analysis data.

**Fig. 8:**
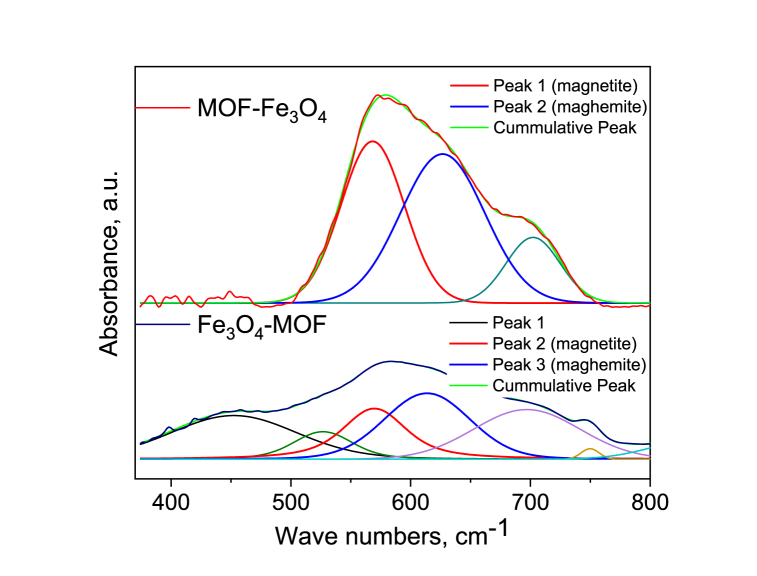
IR-spectra for MOF-Fe_3_O_4_ and Fe_3_O_4_-MOF samples: deconvolution fitting for bond 582 cm^−1^ for MOF-Fe_3_O_4_ and 585 cm^−1^ for Fe_3_O_4_-MOF.

Namduri et al. [59] using FTIR spectroscopy data for quantification of magnetite and maghemite phases in multi-component unknown sample showed that FTIR spectrum of magnetite exhibits two strong infrared absorption bands at 570 cm^−1^ (t1, mode of the tetrahedral and octahedral sites according to Ref. [60] and 390 cm^−1^ (t2). Maghemite has absorption bands at 630 cm^−1^, 590 cm^−1^, and 430 cm^−1^. Thus, the 570 cm^−1^ and 630 cm^−1^ bands, respectively, can be considered the most intense characteristic bands of magnetite and maghemite. The deconvolution of the bands 582 cm^−1^ for MOF-Fe_3_O_4_ and 585 cm^−1^ for Fe_3_O_4_-MOF showed that the bands contained in them can be attributed to magnetite (568.25 ± 1.74 cm^−1^ for MOF-Fe_3_O_4_ and 569.70 ± 8.26 cm^−1^ for Fe_3_O_4_-MOF) and maghemite (626.69 ± 3.12 cm^−1^ for MOF-Fe_3_O_4_ and 613.72 ± 14.54 cm^−1^ for Fe_3_O_4_-MOF) (Fig. 8).

**Fig. 9:**
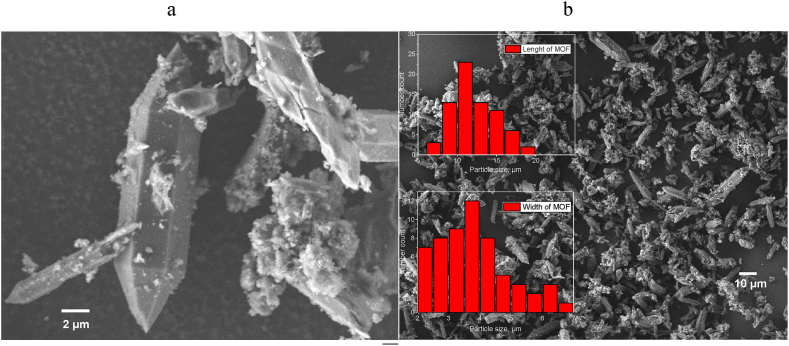
SEM images of MOF at 2 μm (a) and 10 μm (b) resolution, size distribution for MOF length and width (In order to receive statistical particle distributions at least 100 particles were counted per picture; at least three pictures were used for each calculation).

**Fig. 10:**
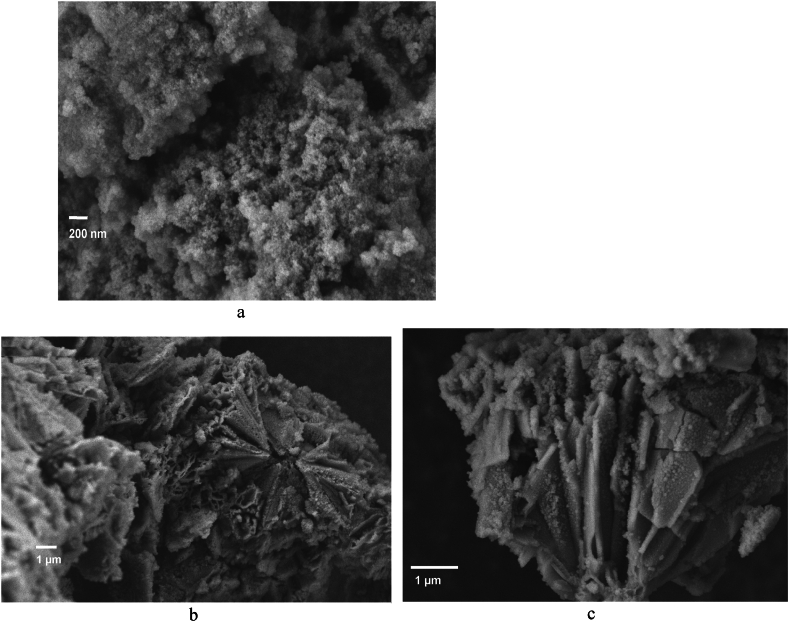
SEM of MOF-Fe_3_O_4_ (a) and Fe_3_O_4_-MOF(*c*,*d*) Besides counting of particle diameters from TEM micrographs (Fig. 11).

The size change of MOFs after functionalization with magnetite was evaluated using TEM and SEM. According to the data of SEM micrographs (Fig. 9, Fig. 10), MOF agglomerates of the MIL-88b type have the shape of an elongated hexagonal bipyramid or rod-like shape, which is often found in the previous research [[74], [75], [76]]. According to statistical processing, the length of the MOF particles was 12.27 μm ± 2.89 μm (Fig. 9c), and the width was 3.84 μm ± 1.13 μm (Fig. 9d), which correlates with the dimensions of MIL-88b obtained in other studies. Thus, Gao et al. [74], reported about the MOF length of 6–8 μm and the particle diameter of 4 μm, and Wang et al. [75], report about the MOF length of 2.8 μm and the particle diameter of 12 nm.

**Fig. 11:**
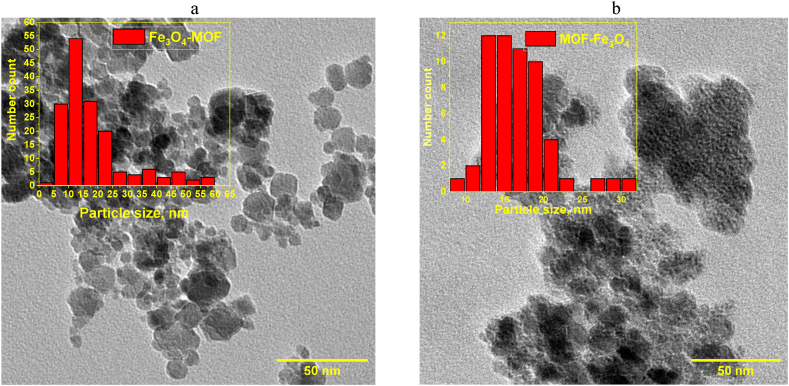
TEM images of samples: (a) for Fe_3_O_4_-MOF with particles distribution; for MOF-Fe_3_O_4_ (b) with particles distribution (In order to receive statistical particle distributions at least 100 particles were counted per picture; at least three pictures were used for each calculation.

Functionalization with magnetite leads to a dramatic change in the morphology of the samples. Thus, for MOF-Fe_3_O_4_ samples (Fig. 10 a), the morphology is close to the morphology of non-functionalized magnetite, which represents spherical particles. Moreover, elemental analysis data also confirms practiclally no MOF into MOF-Fe3O4 sample according to content of C ca. 1 %, which is consistent with our and other data [[77], [78], [79]]. The probable reasons for the destruction of MOFs during synthesis are discussed in the discussion section. At the same time, the Fe_3_O_4_-MOF (Fig. 10 c, d) sample demonstrates a significant change in the morphology of the MOF from pyramidal to lamellar, probably associated with the limitation of the growth of the MOF along one of the magnetite planes.

A decrease in the concentration of MOF in the sample naturally led to a decrease in the specific surface area (SSA) from 220.2 m^2^/g for MOF to 197.8 m^2^/g for Fe_3_O_4_-MOF and 127 m^2^/g for MOF-Fe_3_O_4_ (Table 2). The SSA and the pore diameter of samples determined by N_2_ absorption–desorption technique using Brunauer-Emmett-Teller and Barrett-Joyner-Halenda methods, respectively are presented in Table 2.

**Table 2:** Specific surface area and pore size of samples.

	S_BET,_ m^2^/g	V(BET) pore for P/P_0_ = 0.99, сm^3^/g	BJH	
			V, сm^3^/g	d, nm
MOF	220.2	0.32	0.25	3.59
MOF-Fe_3_O_4_	127.3	0.27	0.23	3.61
Fe_3_O_4_-MOF	197.8	0.29	0.27	8.74

The SSA for the MOF made 220.2 m^2^/g and an average pore diameter of 3.59 nm. The preparation of magnetite in the presence of MOF led to a significant decrease in the surface area to 127.3 m^2^/g, which may be associated with the destruction of MOF under strongly alkaline conditions of synthesis that confirmed by elemental analysis (0.93% C in the MOF-Fe_3_O_4_ sample). On the other hand, the preparation of MOF in the presence of magnetite (Fe_3_O_4_-MOF) slightly reduced in compare with native MOF the surface area to 197.8 m^2^/g at C content of 11.74 %. Interestingly to note, that this method of preparation led to an increase in average pore diameter to 8.74 nm.

**Fig. 12:**
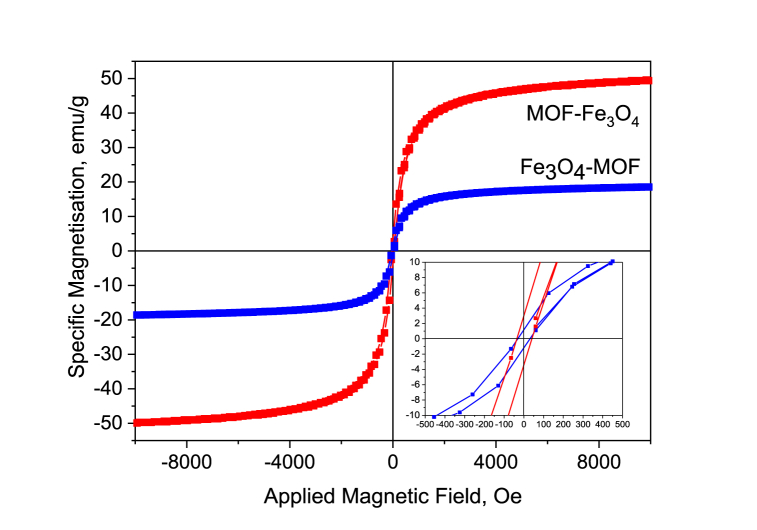
Magnetization curves (room temperature) for MOF modified with Fe_3_O_4_.

The most crucial property of MNPs, which allow a variety of applications, especially in biomedicine, is represented by their ferrimagnetism. As a matter of fact, the application of an external magnetic field makes it possible to concentrate magnetic NPs in the target point, thus reducing likely side effects of iron release. The hysteresis loops for the three different MNPs samples were closed and symmetrical with respect to the origin of the coordinate system as reported in Fig. 12. The shape of the loops indicates the ferrimagnetic features of the material which are prone to their potential application.

Some magnetic characteristics for the magnetite-modified MOF NPs are presented in Table 3. The saturation magnetization σ_s_ of MOF-Fe_3_O_4_ and Fe_3_O_4_-MOF samples were respectively 49.9 and 18.6 emu g^−1^ (Table 3), which is consistent with 62.4 and 42.9% of Fe content respectively according to elemental analysis. The determined saturation magnetization σ_s_ values indicate that NMPs exhibit superparamagnetic properties at room temperature.

**Table 3:** Magnetic properties of samples MOF modified with Fe_3_O_4_.

Sample	σ_s_, G∙cm^3^g^−1^	σ_r_, G∙cm^3^g^−1^	H_c_, Oe
MOF-Fe_3_O_4_	49.9	3.00	35
Fe_3_O_4_-MOF	18.6	1.17	31

In the absence of a magnetic field, all samples showed a similar low residual magnetism ∼±1–3 emu g^−1^ (Table 3) due to magnetic viscosity for superparamagnetic materials [81]. In general, the magnetite-modified MOF MNPs exhibit a saturation magnetization sufficient to control an external magnetic field. For biomedical applications such as drug delivery and hyperthermia a saturation magnetization of ∼8–19 emu g^−1^ is required which according to vibrating-sample magnetometry results the synthesized multifunctional magnetite mesoporous silica nanoparticles coated with a chitosan hydrogelare useful for biomedical applications [[82], [83], [84]]. In many works focused on the preparation of materials for catalyzing ferroptosis the authors have used iron salts as iron ions sources [29, 91, 92]. However, as reported by Huo et al. in Ref. [85], without targeting only 6.95% of MNPs were stored and localized in the 4T1 cancer cell 48 h upon injection.

## 4 Discussion

In this study, the functionalization of MOF with magnetite NPs was carried out to magnetize MOF, which will allow to control the material when exposed to an external magnetic field. In order to select conditions for maximum preservation of the structure and properties of the magnetic complex, this study used two approaches to the functionalization of a known coordination polymer MIL 88B (Fe) with magnetite Fe3O4: (i) by synthesizing magnetite in the presence of pre-synthesized MOF, and vice versa, (ii) by synthesizing MOF in the presence of pre-synthesized magnetite. Both MOFs and magnetite NPs are known to be sensitive to reaction conditions. For example, magnetite NPs are easily oxidized to maghemite [86], which leads to a decrease in saturation magnetization. Thus, in this study, the state of magnetite and MOF was monitored using different methods to obtain a reliable picture of the structure of the components. Control over the preservation of magnetite was carried out by assessing the percentage of magnetite in both synthesis approaches using X-ray phase analysis, Mössbauer spectroscopy and Raman spectroscopy. The use of various methods to quantify of magnetite is justified by the following. While distinguishing between magnetite and maghemite from XRD is quite difficult [48], in Mössbauer spectroscopy, divalent and trivalent iron ions can easily be distinguished due to different isomer shifts [68]. With Raman spectroscopy, magnetite and maghemite NPs can even be accurately differentiated at room temperature. Different phonon modes exist for the inverse spinel configuration of magnetite and the defect spinel structure of maghemite which correspond to different Raman shifts [87,88].

**Fig. 13:**
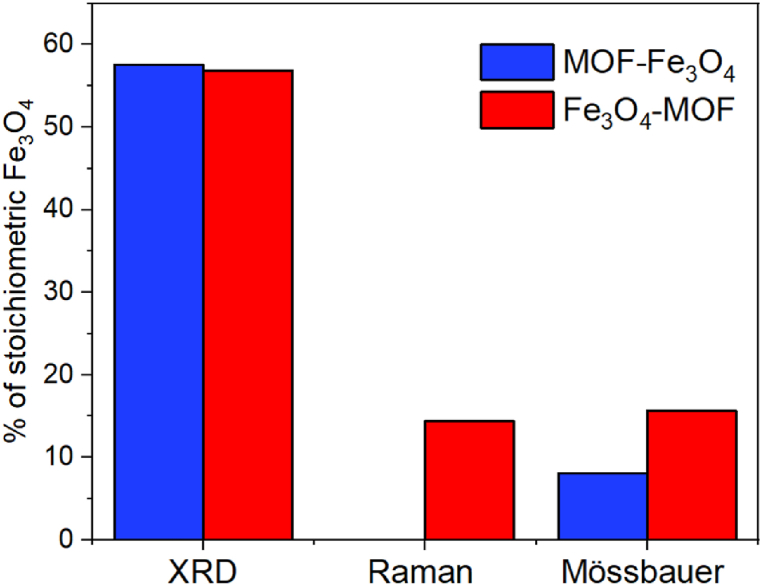
Comparison of % of stoichiometric Fe_3_O_4_ for different samples.

Comparative analysis of magnetite content in magnetic MOF complexes using different methods is presented on Fig. 13.

Thus, the preparation of magnetite in the presence of MOF (MOF-Fe3O4) leads to the complete oxidation of Fe^2+^ ions to Fe^3+^ ions, while Mössbauer spectroscopy data indicate partial retention of Fe^2+^ ions in the core of the sample MOF-Fe_3_O_4_. The approach to synthesis of MOF particles in the presence of magnetite NPs (Fe3O4-MOF) makes it possible to partially retain Fe^2+^ ions both on the surface and in the core of the sample Fe_3_O_4_-MOF leads to particles in a transition state between magnetite and maghemite. This is in good accordance with previous findings [54]. The difference in results may be explained by differences in the nature of the analysis methods used. With Mößbauer spectroscopy one can see the oxidation of the iron ions before the whole crystal changes. With Raman, one observes slightly different distances between iron ions and oxygen ions and with XRD one observes the oxidation at last when everything is completely reoriented in a new lattice. In general, both existing phases of the magnetite-maghemite mixture in both samples allow them to be successfully used to control the magnetic field.

**Fig. 14:**
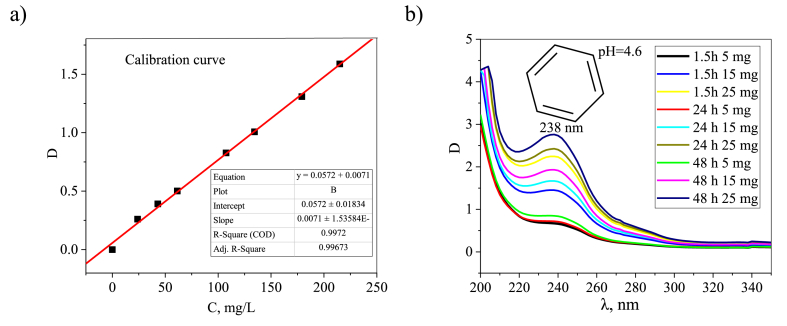
Calibration curve for determining the content of TPA in an aqueous solution (a); UV spectra of solutions after centrifugation at different times and MOF concentrations at pH = 4.6.

Regarding MOF structure, we observe more significant changes in the two synthesis approaches. Heterogeneous MOF systems are capable to degrade in aggressive environments [89]. Degradation is also important for selecting conditions for the synthesis of composite materials with MOFs. The very low content of MOF (according to 1 % C) into MOF-Fe3O4, i.e. during synthesis in situ for magnetite NPs (MOF-Fe_3_O_4_) was a reason to for further study. To study the stability of MIL-88b in different media, buffer solutions were prepared: acetate buffer with pH 4.6 and phosphate buffer with pH 8. The degree of MOF decomposition was determined using a UV spectrometer (SPECS-SSP-705-1 spectrometer, JSC Spectroscopic Systems, Moscow, Russia) based on the peak intensity of 238 nm, corresponding to the electronic transitions of the benzene ring of terephthalic acid (TPA). To calculate the true concentration of TPA, a calibration graph was constructed (Fig. 14). The solubility of TPA is low, so it was converted to the potassium form to obtain calibration dependences. Preliminary studies have shown that conversion to this form does not affect the extinction of the benzene ring. The samples were also kept in distilled water with a resulting pH of 5.6.5, 15, or 25 mg of synthesized MOF were placed in 10 g of the test medium. The tubes were mixed with a shaker for the first 90 min, then centrifuged at 1100 rpm, and the mother solution was examined using UV. When studying the mother liquor in phosphate buffer, the filtrate was subjected to a 10-fold dilution due to high concentrations of TPA. The degree of MOF degradation was determined based on the percentage of TPA in the structure. The stability of the synthesized MOF (MIL-88B) was studied at three pH values.

**Fig. 15:**
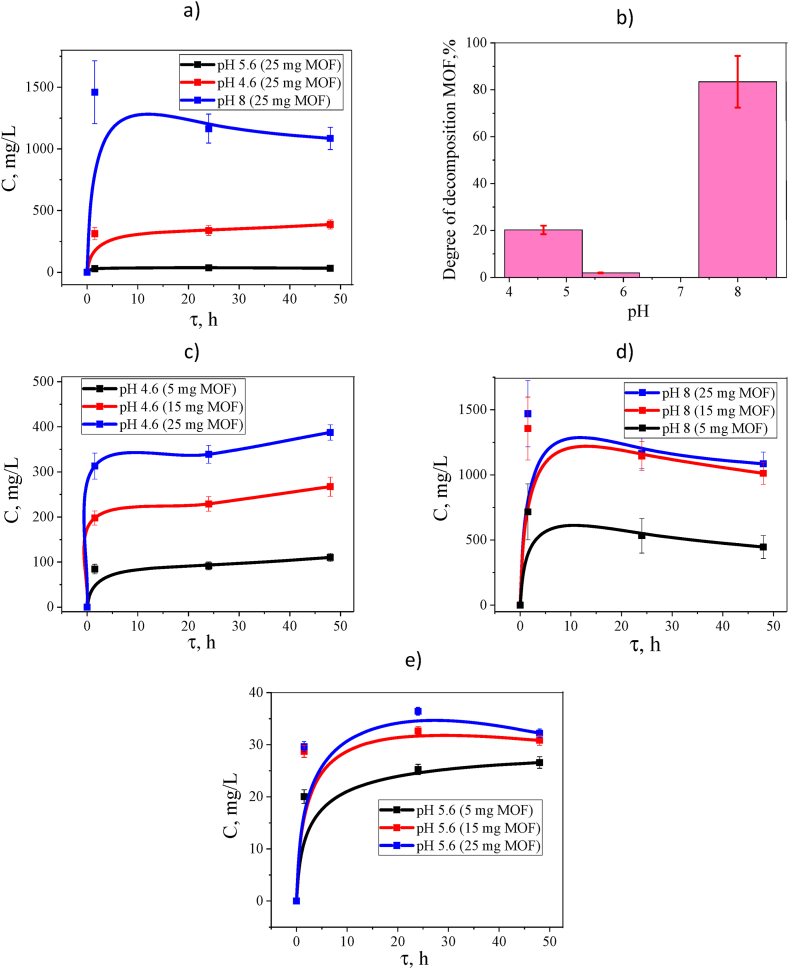
Concentration of released TPA at different pH upon degradation of MOF (MIL-88b) with different masses (a, c, d, e); and degree of degradation of MOF after 90 min of treatment (b).

When MOF is kept in distilled water with pH = 5.6 for two days, no significant increase in TPA concentration is observed (Fig. 15e). The concentration of TPA released from MOF is practically independent of the mass of the initial sample; the maximum release is observed after 1.5 h of exposure. Apparently, in this case, there is an equilibrium release of unreacted TPA physically adsorbed in the pores. At acidic pH (4.6), a release of TPA equivalent to the weight of the sample used is observed (Fig. 15c). 20% of MOF is destroyed already at 2 h of exposure, then the degradation process slows down, but continues. A similar effect was discovered during the study for NH_2_-MIL-88B(Fe) in the pH range from 3.0 to 11.0; the critical value turned out to be pH = 5.0, above which the number of released iron ions tended to zero, and when moving towards more At acidic pH, iron ions were actively released [22]. The influence of the alkaline environment appears to be significant on the synthesized MIL-88b. Thus, it was demonstrated that MOF degrades by 80% already in the first 1.5 h of exposure to phosphate buffer (Fig. 2d). A similar effect on degradation was observed when studying the degradation mechanism of two different iron carboxylate MOFs, MIL-100 (Fe) and MIL-53 (Fe) [90]. The authors observed the formation of α-Fe_2_O_3_ and hematite at elevated temperatures or after changes in pH above 7. The release curves at high concentrations of TPA in an alkaline medium do not show a dependence on the sample weight (above 15 mg), partly this behavior can be explained by the poor solubility of TPA in aqueous media and achieving an equilibrium soluble concentration.

Thus, at alkaline pH, significant degradation of MOFs is observed, releasing linkers. This process results in incomplete inclusion of MOF in the synthesis with in situ obtained magnetite NPs (MOF-Fe_3_O_4_). This highlights the importance of in-depth study of the degradation of each type of MOF under different experimental conditions to develop approaches for preparing composite materials.

### 4.1 Potential application as ferroptosis initiators

To understand the prospects of using these NPs as ferroptosis initiators, we preliminarily studied the concentration of ions released into the solution from the surface/volume of NPs at pH 4.5. UV–Vis spectroscopy was used to demonstrate the kinetics and concentration of the released Fe^2+^ and Fe^3+^ from MOF, Fe_3_O_4_-MOF, MOF-Fe_3_O_4_ (see Supplementary Materials, Fig. S2). The concentrations of iron ions were recalculated per 1 g of the iron. Since the Fenton reaction is a disproportionation reaction of hydrogen peroxide (H_2_O_2_) with further production of a highly reactive hydroxyl radical •OH (E_0_ = 2.80 V), the release of Fe^2+^ and Fe^3+^ was evaluated in the absence/presence of hydrogen peroxide to assess the selectivity of the NPs used (since tumor cells are characterized by an increased content of H_2_O_2_).

**Fig. 16:**
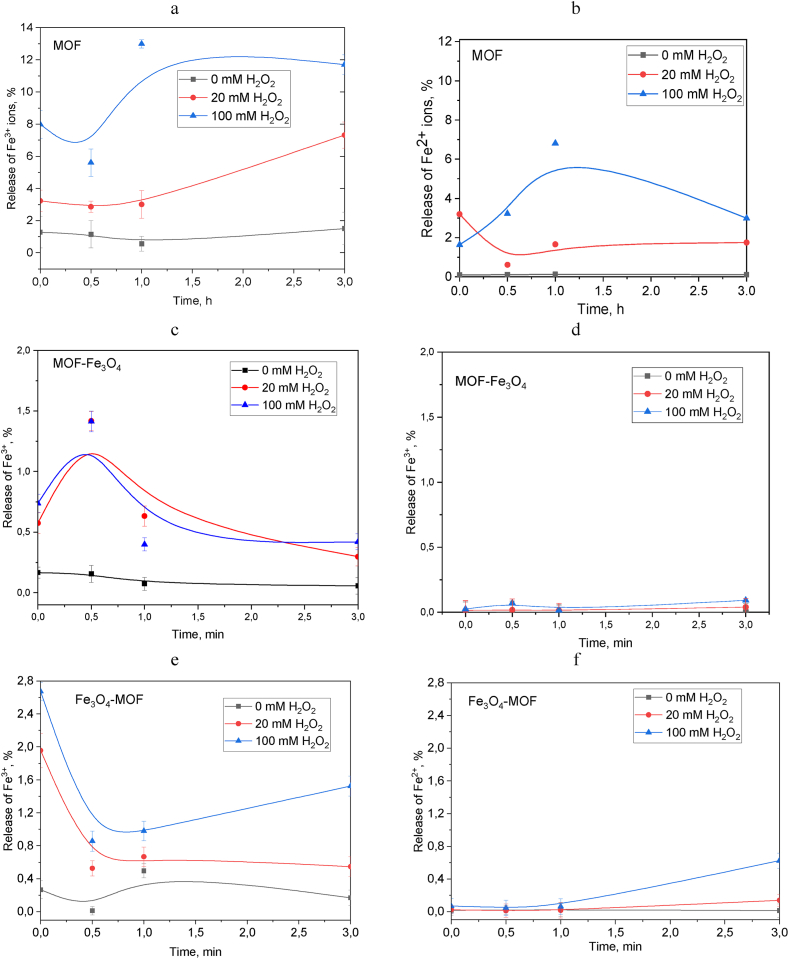
Release of Fe^3+^ and Fe^2+^ from MOF (a, b), MOF-Fe_3_O_4_ (c, d) and Fe_3_O_4_-MOF (e, f) at pH 4.5.

According to Fig. 16 a,b, MOF releases the highest concentration of both Fe^3+^ and Fe^2+^ reaching 12% and 7%, respectively, of the total amount of iron 60 min after the start of measurements. The evaluation was carried out only for iron ions in solution, without taking into account surface iron ions. Functionalization with magnetite leads to a significant decrease in the concentration of released ions to (maximally) 1.3% of Fe^3+^ and 0.1% of Fe^2+^ for MOF-Fe_3_O_4_ (Fig. 16 c, d) and 2.7% of Fe^3+^ and 0.5% of Fe^2+^ for Fe_3_O_4_-MOF (Fig. 16 e, f). A significant decrease in the concentration of ions can be associated with a decrease in the concentration of MOFs in the composition of NPs after functionalization to 0.9% and 33.6% for MOF-Fe_3_O_4_ and Fe_3_O_4_-MOF, while the difference in ions concentration between magnetic composites can also be due to a high content of iron (64.5 %) for the Fe_3_O_4_-MOF sample as compared to the iron concentration in the MOF-Fe_3_O_4_ (42.9%).

## 5 Conclusion

In this work, magnetic materials based on the MIL88b metal-organic framework modified with magnetite were synthesized by two approaches: the preparation of magnetite in the presence of MOF and the preparation of MOF in the presence of magnetite. Analysis of the microstructure and comparison of the results of XRD, Raman and Mössbauer spectroscopy showed that both iron oxide-containing samples are a mixture of two iron oxides, magnetite and maghemite. It has been shown that the production of magnetite in the presence of MOF (MOF-Fe_3_O_4_) leads to the complete transformation of magnetite into maghemite on the nanoparticle surface, while the production of MOF in the presence of magnetite (Fe_3_O_4_-MOF) allows partial retention of Fe^2+^ ions both on the surface and in the core of the sample. The presence of Fe^2+^ ions should favorably affect the rate of the Fenton reaction, which will be assessed in our next work. Also the preparation of MOF in the presence of magnetite (MOF-Fe_3_O_4_) leads to the production of materials with a high content of MOF in the composition (11.74% С for Fe_3_O_4_-MOF compared to 0.93% for MOF-Fe_3_O_4_). A dramatic decrease in the concentration of MOF occurred due to the destruction of the metal-organic framework under alkaline conditions, which was shown by us. A preliminary assessment of the samples as sources of iron ions for initiating the Fenton reaction showed that the most promising materials are samples with a high content of MOF and Fe^2+^ ions (MOF and Fe_3_O_4_-MOF). The application of these samples in the Fenton reaction is object for our future research.

## Funding

This research was funded by 10.13039/501100006769Russian Science Foundation, grant number 22-73-10222. Part of analysis was supported by Austrian Academic Exchange Service (OeAD) MPC-2022-02194. Marco Reindl was trained within the frame of the PhD program in Molecular Medicine.

## Data availability statement

The data is available upon request from the corresponding author.

## CRediT authorship contribution statement

**Lyubov Bondarenko:** Writing – original draft, Project administration, Methodology, Investigation, Formal analysis, Data curation, Conceptualization. **Rose Baimuratova:** Writing – review & editing, Investigation, Formal analysis. **Marco Reindl:** Writing – review & editing, Data curation. **Verena Zach:** Writing – review & editing, Data curation. **Artur Dzeranov:** Writing – review & editing, Formal analysis, Data curation. **Denis Pankratov:** Writing – review & editing, Formal analysis, Data curation. **Kamila Kydralieva:** Writing – review & editing, Supervision, Resources, Project administration, Conceptualization. **Gulzhian Dzhardimalieva:** Writing – review & editing, Formal analysis, Data curation. **Dagmar Kolb:** Writing – review & editing, Methodology, Data curation. **Friedrich E. Wagner:** Methodology, Investigation, Data curation. **Sebastian P. Schwaminger:** Writing – review & editing, Supervision, Methodology, Funding acquisition, Conceptualization.

## Declaration of competing interest

The authors declare that they have no known competing financial interests or personal relationships that could have appeared to influence the work reported in this paper.
