# Supplementary material for: Dramatic change in the properties of magnetite-modified MOF particles depending on the synthesis approach
Source: Heliyon. 2024 Mar 14;10(6):e27640. doi: 10.1016/j.heliyon.2024.e27640 (PMC10958221; doi:10.1016/j.heliyon.2024.e27640)
Supplement: Multimedia component 1 [file mmc1.docx]

Scherrer equation for spherically-shaped particles: (S1)

D (nm) = $\frac{0.89\cdot\lambda}{\Delta2\theta\cdot cos(\theta)}$

Where λ - X-ray source wavelength, nm

Δ2θ - peak width at half its height, °

Bragg equation for cubic system: (S2)

a= $\sqrt{\frac{\lambda^{2}}{4\cdot{sin}^{2}\theta}(h^{2}+k^{2}+l^{2})}$

Where λ - X-ray source wavelength, nm

hkl - Miller indices





Fig. S1. XRD for MOF and the calculated CCDC 2088535 (Cu-Kα radiation, λ = 1.5409 Å)

| a) | b) |
| --- | --- |
| 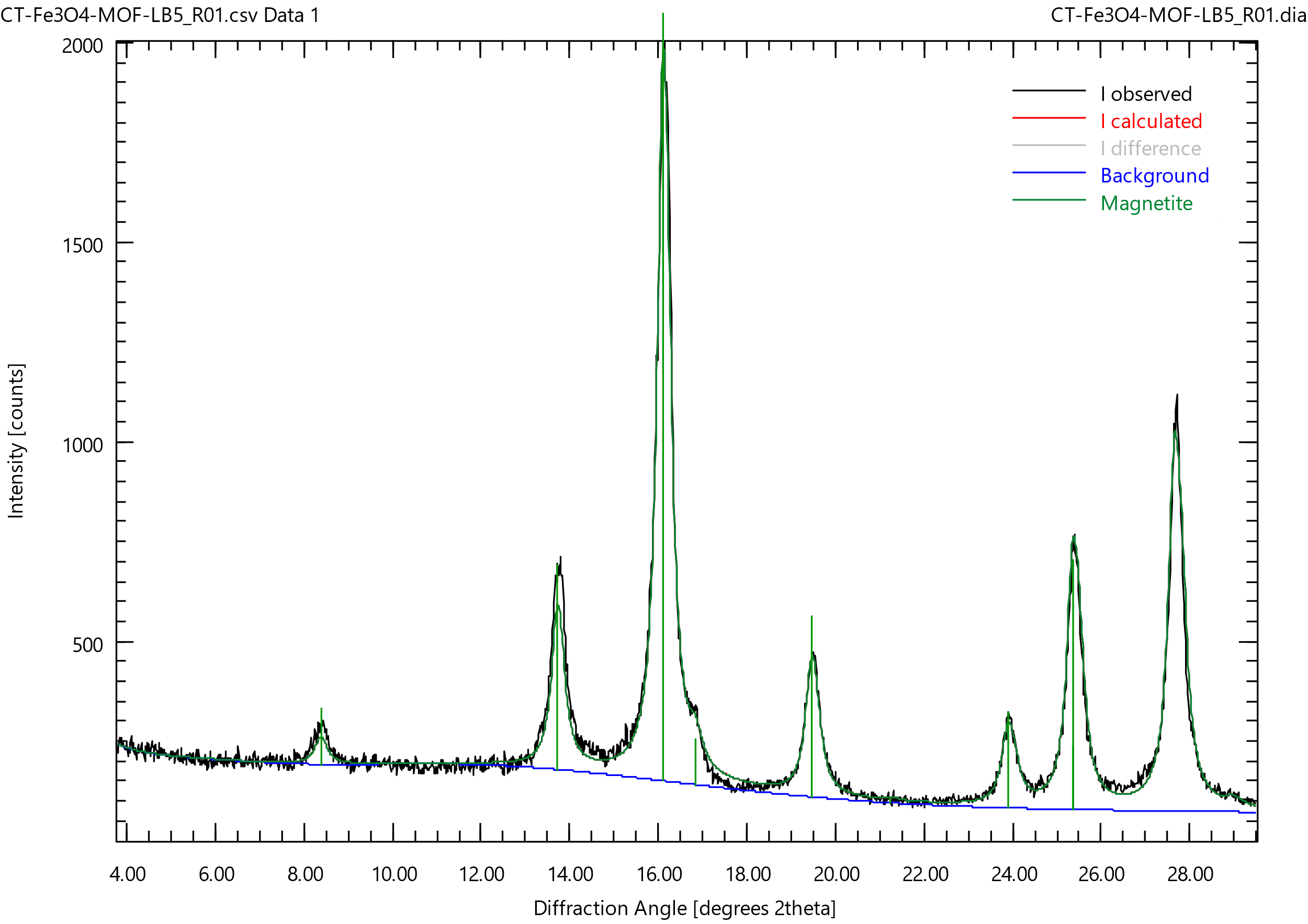 | 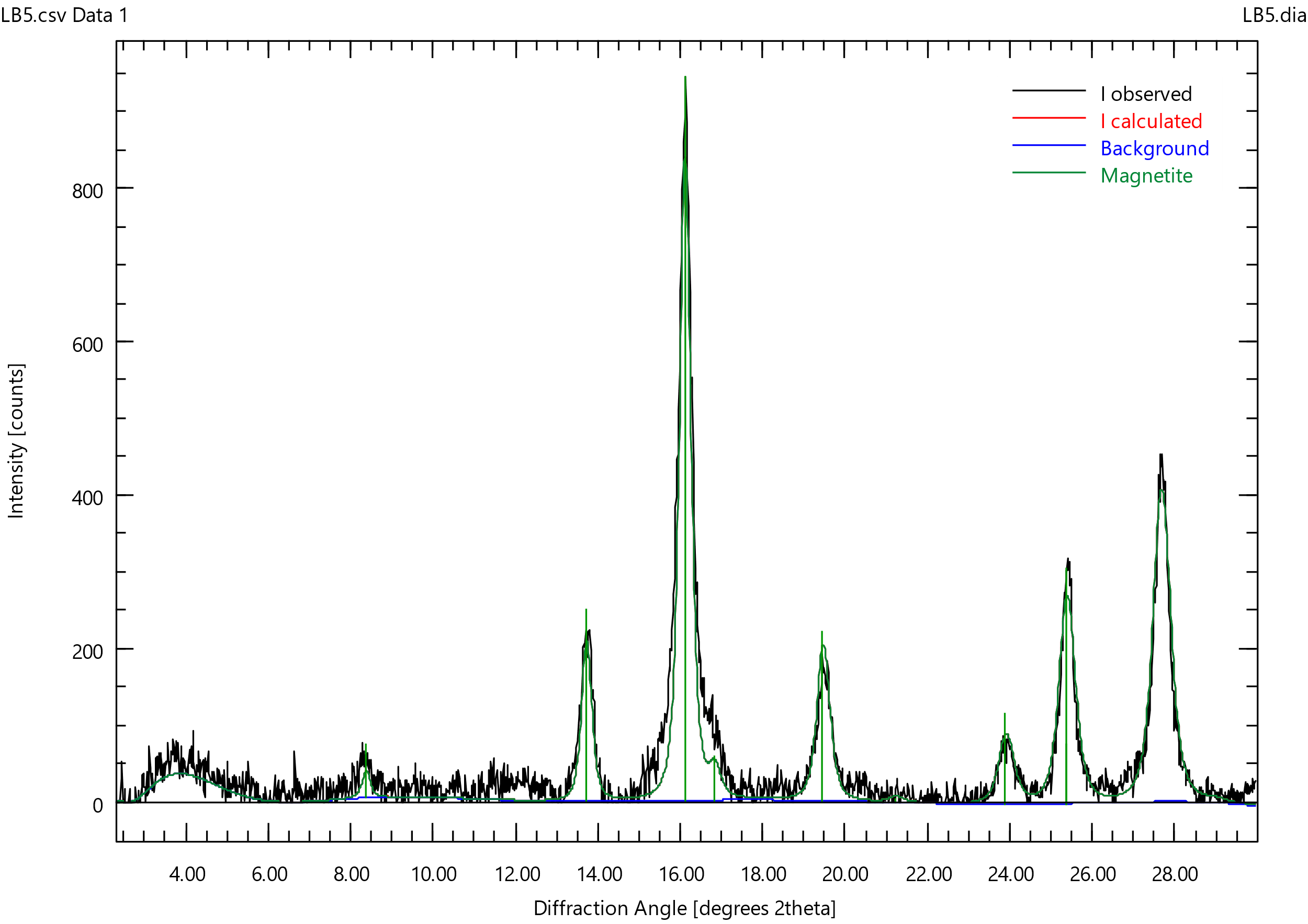 |

Fig. S2. Experimental and calculated (Rietveld) XRD patterns of MOF-Fe3O4 (a) and Fe3O4-MOF (b) with background lines, difference and standard magnetite spectra.

| 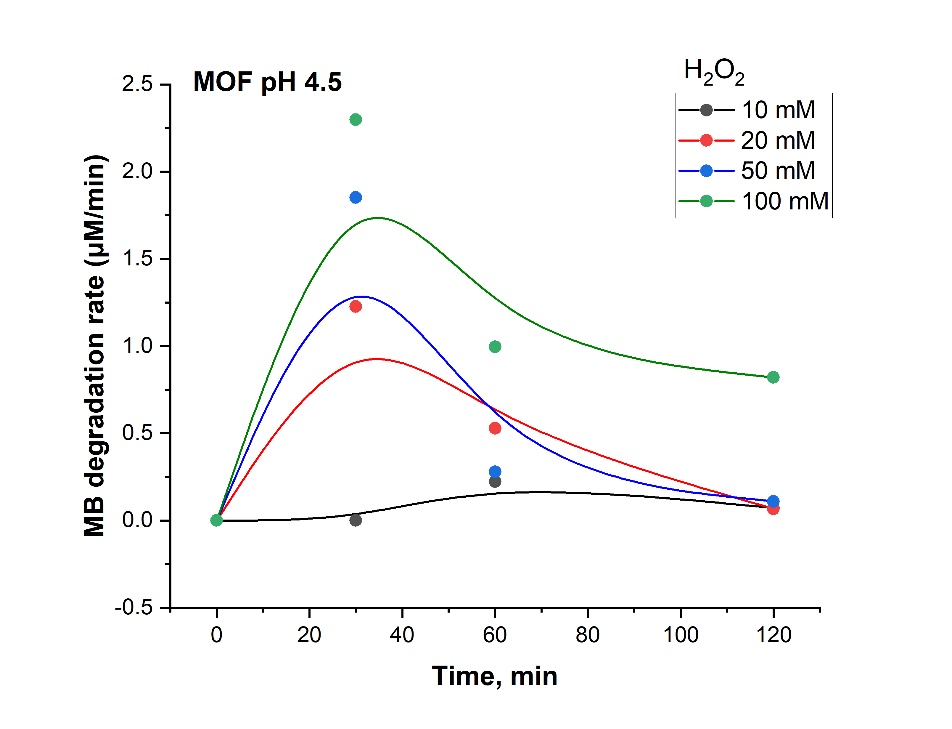 | |
| --- | --- |
| 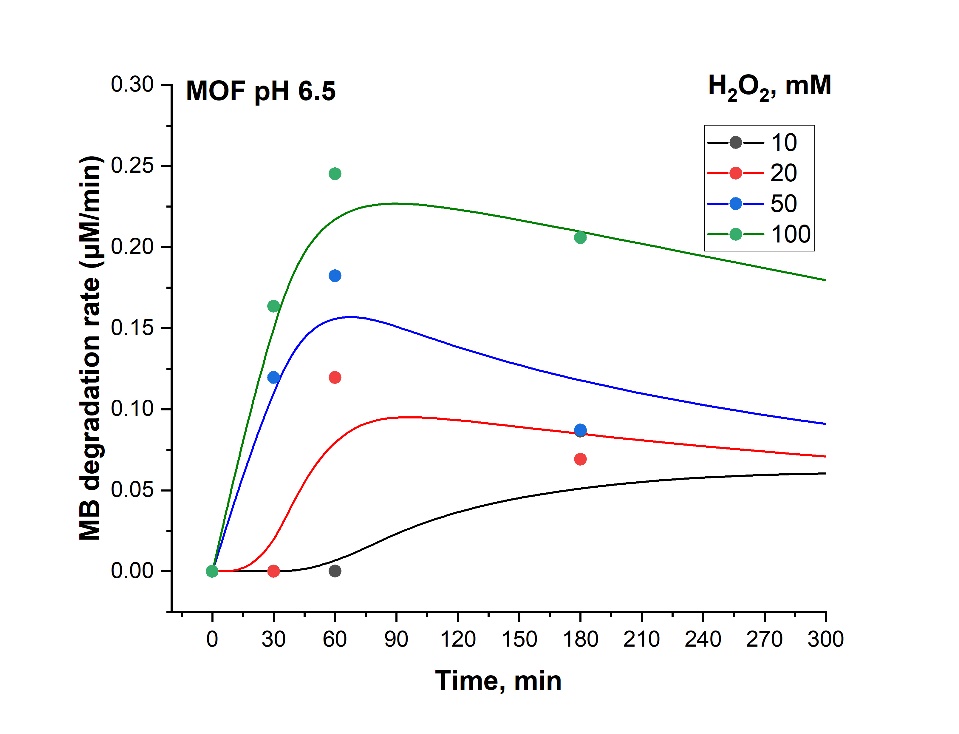 | |
| 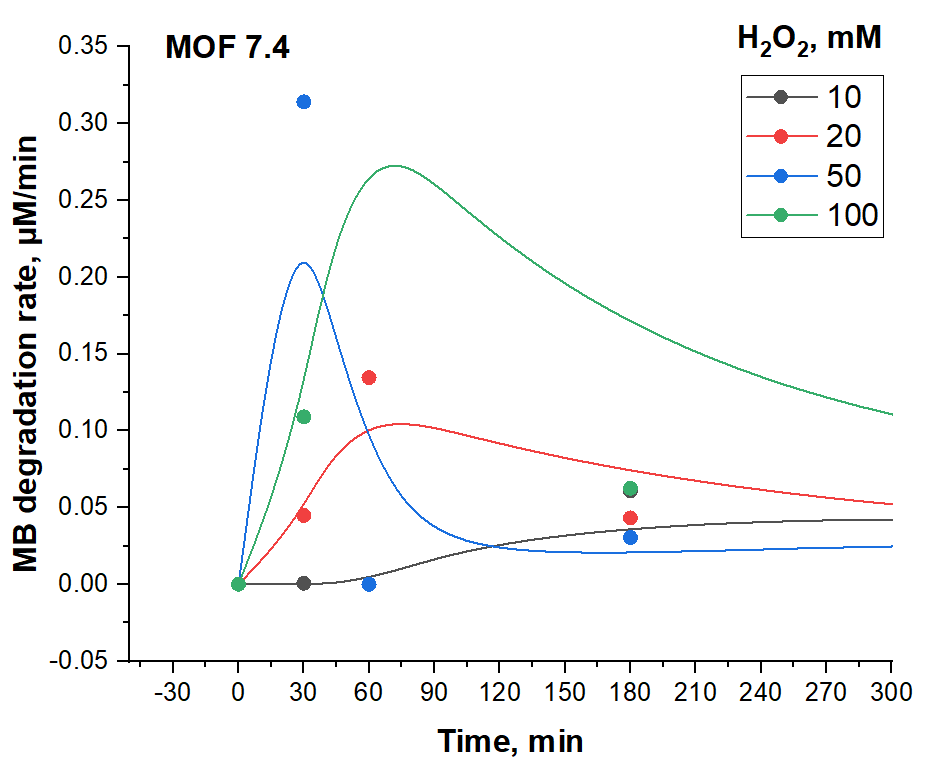 | |
| 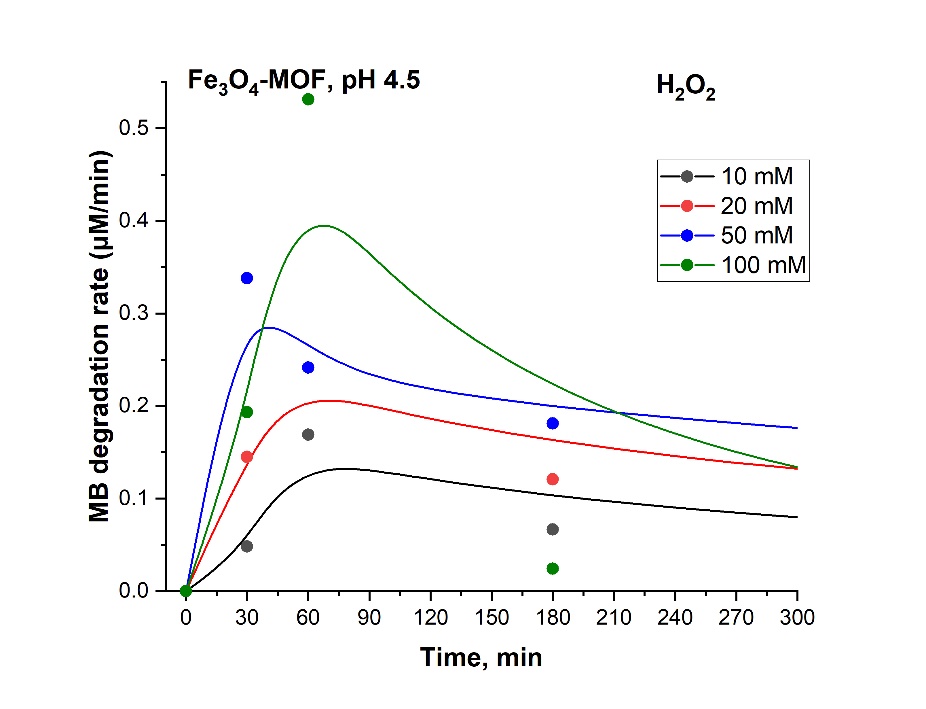 | 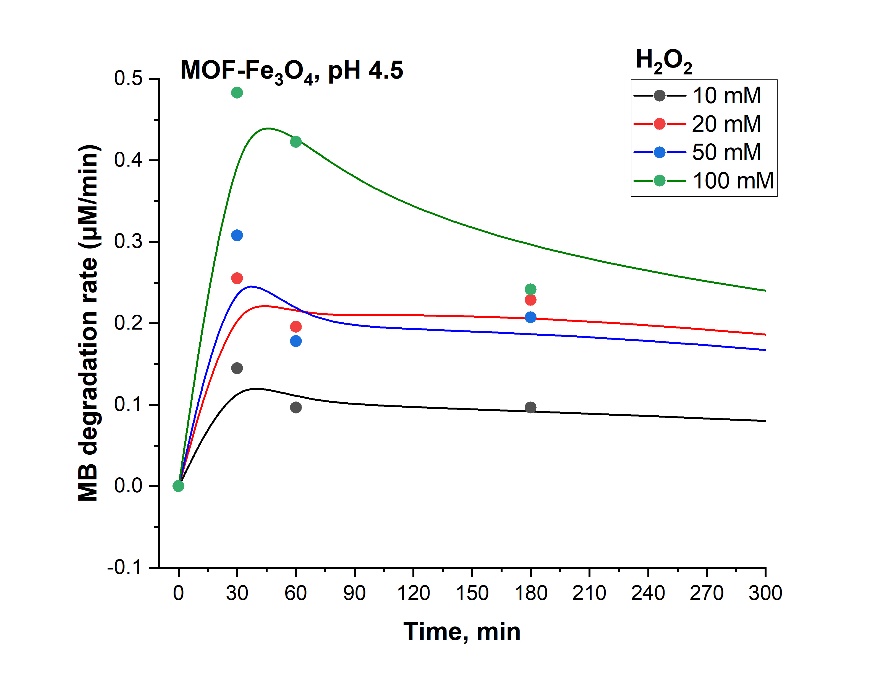 |
| 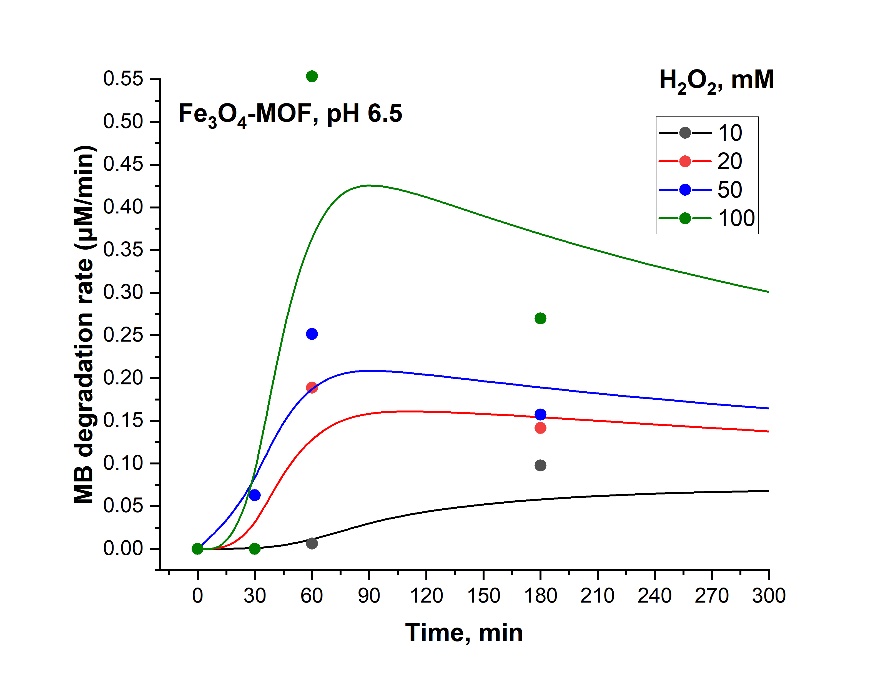 | 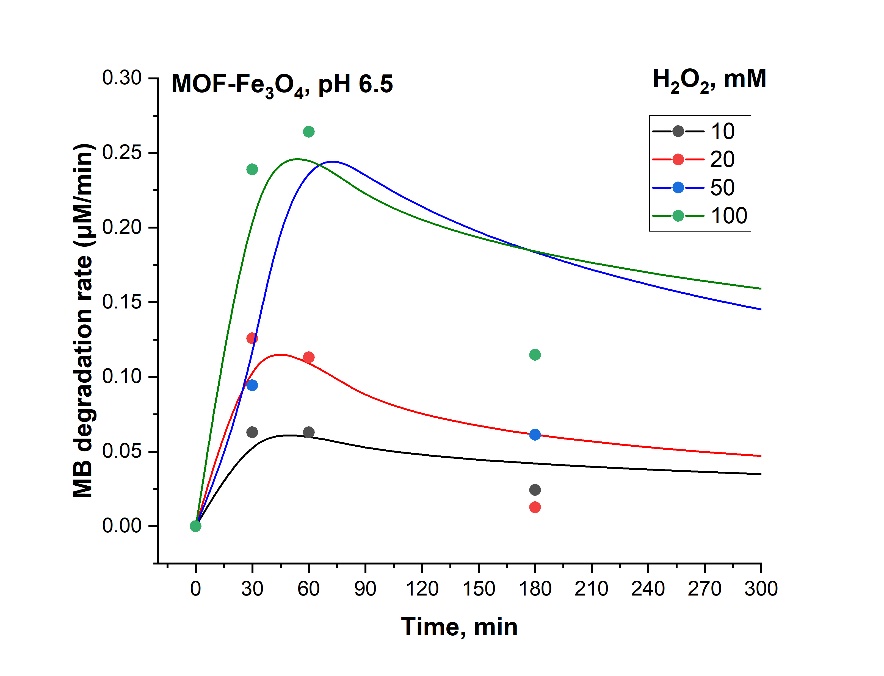 |
| 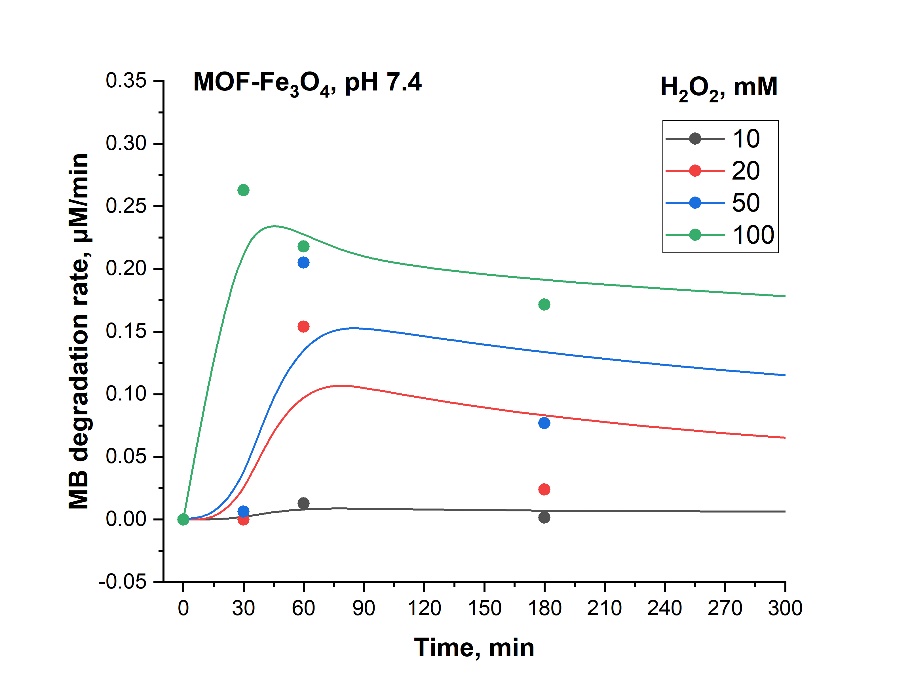 | 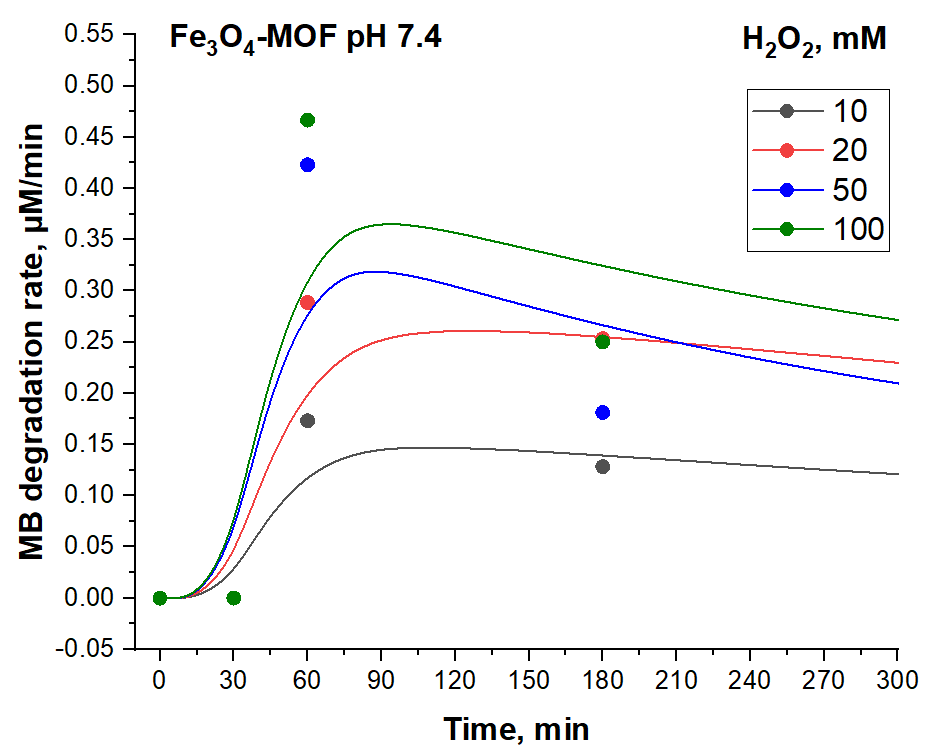 |

Fig. S3. Curves of Methylene Blue Degradation Rates in the Presence of MOF and MOF-Modified Species
